# Integrating positive psychology into undergraduate oncology education: development and evaluation of the 4C-HOPE teaching model

**DOI:** 10.3389/fmed.2026.1914104

**Published:** 2026-09-09

**Authors:** Minling Liu, Chengrui Zhong, Tingwei Li, Huiru Dai, Jiancheng Wang, Shuo Fang

**Affiliations:** 1 Department of Oncology, The Seventh Affiliated Hospital of Sun Yat-sen University, Shenzhen, China; 2 Department of Hepatobiliary and Pancreatic Surgery, The Seventh Affiliated Hospital of Sun Yat-sen University, Shenzhen, China; 3 Scientific Research Center, The Seventh Affiliated Hospital of Sun Yat-sen University, Shenzhen, China; 4 Academic Affairs Department, The Seventh Affiliated Hospital of Sun Yat-sen University, Shenzhen, China

**Keywords:** 4C-HOPE teaching model, hepatocellular carcinoma, medical education, oncology education, positive psychology

## Abstract

**Background:**

Oncology education often focuses on disease burden and mortality, which may reduce students’ learning interest and reinforce negative perceptions of cancer. Although positive psychology has shown educational benefits in promoting motivation, resilience, and professional development, its integration into oncology education remains insufficiently explored.

**Objective:**

To evaluate the effectiveness of a positive psychology–integrated oncology teaching model (4C-HOPE) in undergraduate medical education.

**Methods:**

Sixty-eight undergraduate medical students at Sun Yat-sen University participated in the 4C-HOPE teaching intervention during the hepatocellular carcinoma (HCC) chapter of their oncology course. Knowledge tests and questionnaires were administered before and after the intervention to assess educational outcomes. Student satisfaction and subsequent clinical clerkship performance were also evaluated.

**Results:**

Knowledge test scores improved significantly, with the median score increasing from 60 to 100 (*P* < 0.001). The proportion of students reporting strong interest in learning about HCC increased from 50.0% to 85.0% (*P* < 0.001). Agreement that physicians should provide holistic care increased from 89.7% to 95.0% (*P* = 0.480), while willingness to apply positive psychology-based communication skills increased from 60.3% to 77.5% (*P* = 0.089). Overall, 87.5% of students expressed high satisfaction with the teaching model. During subsequent clerkships, students exposed to the 4C-HOPE model achieved higher examination scores than controls (81.49 ± 7.87 vs. 77.45 ± 8.58, *P* = 0.014).

**Conclusions:**

The 4C-HOPE teaching model significantly improved knowledge acquisition and learning interest among undergraduate medical students, with favorable trends in holistic care awareness and communication willingness. The sustained benefits observed in clerkship performance further support its pedagogical value. This model offers a feasible framework for fostering both academic learning and professional development in oncology education.

## 1 Introduction

Oncology education presents unique challenges compared with many other medical disciplines because students are frequently exposed to patients facing life-threatening illness, poor prognosis, suffering, and death. Such experiences require not only clinical knowledge but also psychological resilience, empathy, and a well-developed professional identity to provide patient-centered care. A range of learner-centered educational strategies has been introduced into undergraduate oncology education (1–3). Problem-based learning and case-based learning have been widely adopted to promote active learning, strengthen the integration of theoretical knowledge with clinical practice, and improve students’ clinical reasoning, problem-solving abilities, and teamwork skills (4, 5). Similarly, multidisciplinary team-based teaching has been used to integrate knowledge across disciplines and facilitate students’ understanding of comprehensive cancer management through multidisciplinary case discussions (6, 7). Standardized patient-based teaching has been widely adopted in undergraduate oncology education, particularly for developing communication skills (8). Collectively, these educational innovations have substantially enhanced students’ knowledge acquisition, clinical competence, and collaborative decision-making.

However, comparatively less attention has been paid to students’ psychological resilience, professional identity formation, and capacity for patient-centered care. In a large multicenter study, over half of patients with five common cancers were diagnosed at late stages (III-IV) (9). A large US population-based study demonstrated that 14-year cause-specific mortality rates reached 45.8% for stage III and 77.5% for stage IV cancers (10). Moreover, some malignancies have a poor prognosis even when diagnosed at an early stage. For example, the estimated cure fraction for stage I–II hepatocellular carcinoma (HCC) is only 32% (10). Consequently, oncology trainees may frequently encounter patients experiencing suffering, treatment uncertainty, and death (11). Compounding this challenge, undergraduate medical students generally have limited experience managing severe illness and end-of-life issues (12). Taken together, these realities underscore that oncology curricula should move beyond purely biomedical instruction to foster learners’ psychological wellbeing and sustained professional maturation.

Positive psychology offers a promising framework for addressing these educational needs. By emphasizing strengths, resilience, meaning, and hope, it has been associated with improvements in psychological well-being and socio-emotional competence among students (13, 14). In medical education, positive psychology–based approaches have also been linked to greater empathy, resilience, and professional identity development (15). Previous studies have shown that oncology healthcare professionals rely heavily on positive coping strategies and emotional regulation to maintain professional well-being and effective patient care (16). However, to our knowledge, no educational model has systematically integrated positive psychology into undergraduate oncology education.

Drawing on these principles, we developed the 4C-HOPE teaching model, which comprises four sequential stages (Control, Compassion, Confidence, and Calling), and incorporated it into the undergraduate oncology course to help students build positive psychological resources such as hope, resilience, and meaning. This study evaluated its educational impact on learning outcomes, student interest, professional identity formation, and preparedness for future clinical practice, hypothesizing that this approach would enhance both academic learning and professional development.

## 2 Materials and methods

### 2.1 Study participants

A total of 68 undergraduate clinical medicine students (Classes 4–6, 2022 cohort) at Sun Yat-sen University participated in the 4C-HOPE teaching intervention during the HCC chapter of their oncology course on October 23, 2025. HCC was selected as the intervention scenario because it represents a malignancy associated with poor prognosis, with 5-year overall survival rates of 56.1%, 35.3%, 10.7%, and 6.2% for the American Joint Committee on Cancer (AJCC) stage I-IV disease, respectively, and 56.4%, 29.5%, 9.6%, and 3.8% for the Barcelona Clinic Liver Cancer (BCLC) stages A-D, respectively (17). Beyond its unfavorable prognosis, patients with HCC frequently experience complex symptom clusters, including fatigue, pain, appetite loss, sleep disturbance, and emotional distress, which negatively affect quality of life and daily functioning (18, 19).

### 2.2 Teaching methods

**Figure 1:**
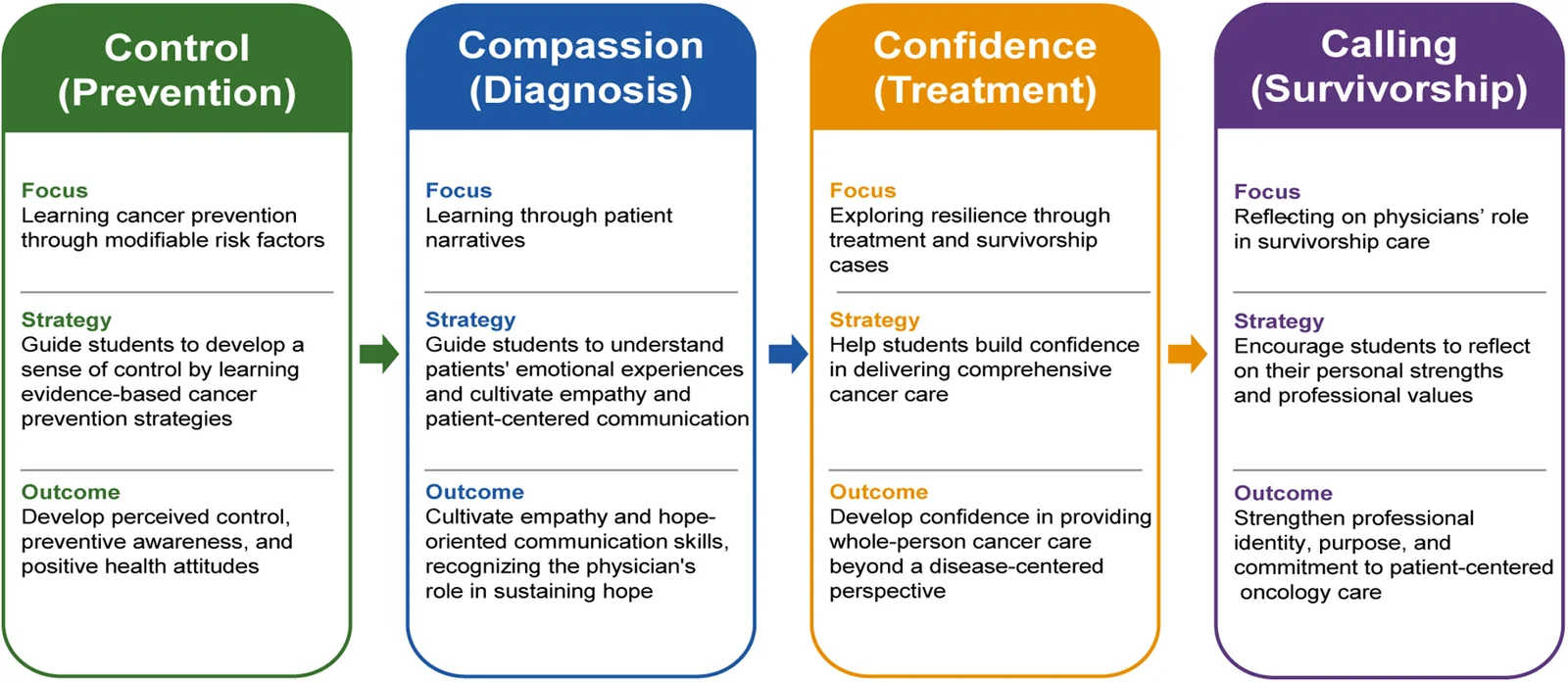
The 4C-HOPE teaching model. Patient-centered oncology scenarios were used as educational resources to cultivate medical students’ positive psychological resources and professional competencies across the cancer care continuum.

The 4C-HOPE teaching model was developed based on positive psychology principles, informed by Seligman's Positive emotion, Engagement, Relationships, Meaning, and Accomplishment (PERMA) model of well-being (20) and Fredrickson's broaden-and-build theory of positive emotions (21, 22) (Table 1), and implemented in the HCC chapter of the undergraduate oncology course (Figure 1). The model comprised four sequential stages—Control, Compassion, Confidence, and Calling—aligned with key phases of the HCC care continuum.

**Table 1:** Theoretical framework of the 4C-HOPE teaching model based on positive psychology.

4C-HOPE component	Positive psychology construct	Theoretical basis
Control	Perceived control/competence	PERMA (Accomplishment, Engagement)
Compassion	Empathy/positive relationships	PERMA (Relationships)
Confidence	Hope/resilience	Broaden-and-Build Theory of Positive Emotions
Calling	Meaning/purpose	PERMA (Meaning)

During the Control stage, which corresponds to the prevention phase, teaching focused on modifiable risk factors for HCC, including hepatitis B vaccination, alcohol cessation, and healthy lifestyle behaviors. Through case-based discussion and preventive medicine scenarios, students were guided to recognize cancer prevention as an achievable and controllable process, thereby strengthening their own perceived control and confidence in promoting health behaviors among future patients-an outcome aligned with the accomplishment dimension of PERMA. The Compassion stage addressed the diagnostic phase of HCC care. Patient illness narratives describing the psychological journey from diagnostic distress to adaptation and hope reconstruction were incorporated as educational materials. These narratives were used not to provide psychological intervention for patients, but to facilitate students’ reflection on patients’ emotional experiences, enhance empathy, and strengthen their ability to establish therapeutic relationships with patients-an approach aligned with the PERMA dimension of relationships. In the Confidence stage, corresponding to the treatment phase, students examined representative cases highlighting treatment resilience, recent therapeutic advances, and quality-of-life improvements achieved through palliative care. Consistent with Fredrickson's broaden-and-build theory, these activities were designed to broaden students’ perspectives beyond prognosis and mortality while building psychological resources such as hope and resilience. Finally, the Calling stage focused on the prognosis and follow-up phase, including long-term follow-up, recurrence surveillance, and survivorship care. Through reflective exercises, students explored how physicians can provide sustained biopsychosocial support and maintain hope throughout the cancer care continuum, thereby cultivating professional qualities such as resilience, responsibility, and communication skills, and finding deeper purpose in their professional roles—an outcome aligned with the PERMA dimension of meaning.

### 2.3 Outcome measures

Data were collected through the Wenjuanxing platform, which was used to administer both the knowledge tests and the online questionnaires. Students were free to choose whether to participate, without any consequences for their course grades. All data were collected anonymously and could not be traced back to individual participants, which may have encouraged students to provide honest responses. However, this anonymous approach also precluded the identification of non-respondents and the use of follow-up reminders. Students were informed of the study by the course instructor at the beginning of the session. Prior to data collection, they received a thorough verbal explanation of the study’s purpose, the voluntary nature of their participation, and the anonymization of all data, and were explicitly informed that completion of the questionnaire would be taken as documented consent to participate. To minimize student burden, the pre-intervention questionnaire was distributed one hour before the class for completion; the post-intervention questionnaire was distributed immediately at the end of the class, and students could either scan the Quick Response (QR) code on site or take a photo of it to complete in three days. The study was approved by the Ethics Committee of our institution (Approval No. KY-2026-247-01).

#### 2.3.1 Baseline perceptions and expectations

Baseline data were collected prior to the intervention, including: (1) students’ attitudes toward oncology learning; (2) perceived helplessness when confronting HCC (5-point Likert scale, 1 = very low, 5 = very high); and (3) expectations regarding the integration of positive psychology into oncology education.

#### 2.3.2 Learning outcomes

Professional knowledge was assessed using identical five-item multiple-choice tests administered before and after the intervention (20 points per item; total score range: 0–100; Supplementary Material 1). Students also rated the extent to which the 4C-HOPE model supported their learning using a 5-point Likert scale (1 = not helpful at all, 5 = extremely helpful).

#### 2.3.3 Learning interest and professional development

Learning interest in HCC was measured using a 5-point Likert scale, assessing changes before and after the intervention. Professional identity formation was evaluated through questions examining students’ perceptions of the physician's role in caring for patients with advanced HCC. Willingness to apply positive psychology-based communication strategies in future interactions with patients with cancer, using a 5-point Likert scale.

#### 2.3.4 Student satisfaction

Overall satisfaction with the 4C-HOPE teaching model was evaluated using a 5-point Likert scale (1 = very dissatisfied, 5 = very satisfied). An open-ended question was also included to collect student feedback on course improvement.

#### 2.3.5 Long-term follow-up

Between November 2025 and April 2026, following completion of the 4C-HOPE classroom intervention, students in the 2022 cohort completed a 1-week oncology clerkship rotation, with the theoretical examination administered on the final day of the rotation. The examination covered several common cancers and included multiple-choice, short-answer, and case-based questions. All examination papers were centrally developed by the Oncology Teaching Office of Sun Yat-sen University and were not disclosed to students before the examination. To ensure comparable difficulty across different examination weeks, the teaching office used a standardized examination framework for paper development. Of the 140 students in the 2022 cohort who completed their oncology clerkship at our hospital, 36 were from the 68-student 4C-HOPE intervention cohort, whereas the remaining 104 students were from Classes 1–3 and 7–12 and received the conventional oncology curriculum without the 4C-HOPE intervention. Clerkship examination scores were subsequently compared between these two groups.

### 2.4 Statistical analysis

All statistical analyses were performed using SPSS 27.0 software. Cronbach's alpha was used to assess questionnaire reliability, with McDonald’s omega (*ω*) also calculated for confirmation. Because the questionnaires were collected anonymously, individual-level matching between pre-intervention and post-intervention responses was not possible. Therefore, the analyses were performed to compare differences between the two assessment time points at the group level rather than to evaluate individual-level changes over time. Statistical approaches were determined by data type: (1) Continuous data: Normality was assessed using the Shapiro–Wilk test. Normally distributed data were presented as mean ± standard deviation and compared using independent samples *t*-test. Non-normally distributed data were presented as median (M) and interquartile range (*P*25, *P*75) and compared using the Mann–Whitney *U* test. (2) Ordinal data: Presented as number (percentage) and compared using the Mann–Whitney *U* rank-sum test. (3) Categorical data: Presented as number (percentage) and compared using Pearson's chi-square test, with Fisher's exact test used when expected frequencies were <5. A two-sided *P*-value < 0.05 was considered statistically significant. Given the exploratory nature of this study and the limited number of pre-specified comparisons, we did not apply corrections for multiple comparisons.

## 3 Results

### 3.1 Questionnaire response rate and reliability

Sixty-eight students completed the pre-intervention questionnaire, and 40 completed the post-intervention questionnaire. The primary reason for non-completion of the post-intervention survey was class schedule constraints—some students left immediately after the session for subsequent classes or meals and therefore did not complete the questionnaire. The questionnaire demonstrated acceptable internal consistency, with Cronbach's *α* values of 0.621 and 0.923 for the pre- and post-intervention questionnaires, respectively. McDonald’s *ω* yielded consistent results (*ω* = 0.625 and 0.944, respectively).

### 3.2 Baseline perceptions and expectations

Baseline findings indicated that students experienced considerable emotional challenges when learning oncology and expressed strong interest in the integration of positive psychology into oncology education.

As shown in Table 2, the most common attitude toward oncology learning was a calm and detached approach focused primarily on knowledge acquisition (36.76%), followed by curiosity and exploration (29.41%). Only 22.06% of students reported a positive sense of professional mission, whereas 11.76% described feelings of anxiety or distress related to patient suffering and poor prognoses. The mean perceived helplessness score when confronting HCC was 4.21 ± 0.84 on a 5-point scale, indicating a substantial sense of helplessness among students before the intervention.

**Table 2:** Baseline attitudes toward oncology learning (*N* = 68).

Attitude toward oncology learning	*n* (%)
Calm and detached: Focused primarily on acquiring medical knowledge while maintaining emotional distance	25 (36.76)
Curiosity and exploration: Interested in disease mechanisms, diagnosis, and advances in cancer treatment	20 (29.41)
Positivity and mission: Motivated to master oncology knowledge despite disease severity in order to help patients	15 (22.06)
Anxiety and depression: Frequently experienced distress when confronted with patient suffering and poor prognoses	8 (11.76)

Students also reported clear expectations regarding the incorporation of positive psychology into oncology education (Table 3). The most frequently identified need was a better understanding of hope, survivorship, and palliative care in oncology practice (80.88%), followed by enhancing learning motivation through positive emotions (72.06%). Approximately two-thirds of students expected positive psychology–based teaching to strengthen their professional identity by helping them learn how to instill confidence and hope in patients (69.12%) and to reduce anxiety associated with oncology learning while maintaining interest (64.71%).

**Table 3:** Students’ expectations regarding the integration of positive psychology into oncology education (*N* = 68).

Expected support from positive psychology in oncology learning	Needed, *n* (%)	Not Needed, *n* (%)
Enhancing understanding of hope and the value of survivorship and palliative care in oncology practice	55 (80.88)	13 (19.12)
Promoting learning motivation and improving learning effectiveness through positive emotions	49 (72.06)	19 (27.94)
Strengthening professional identity by developing the ability to instill confidence and hope in patients	47 (69.12)	21 (30.88)
Reducing anxiety and emotional distress associated with oncology learning while maintaining learning interest	44 (64.71)	24 (35.29)

### 3.3 Learning outcomes

**Figure 2:**
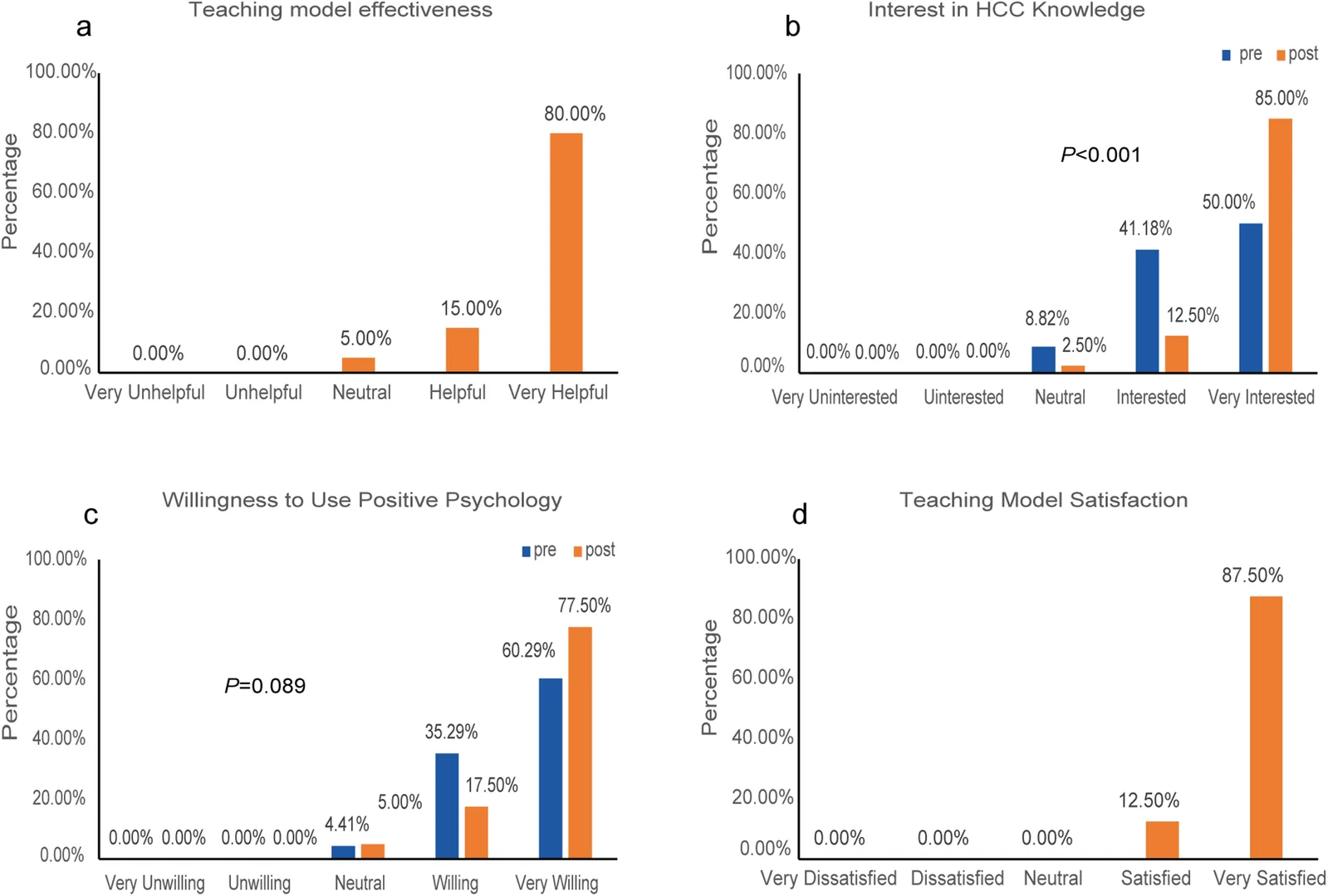
Student evaluation of the teaching model. **(a)** Perceived helpfulness of the teaching model in mastering professional knowledge (*N* = 40). **(b)** Changes in learning interest in HCC knowledge (pre: *N* = 68; post: *N* = 40). **(c)** Willingness to apply positive psychology communication skills in future clinical practice (pre: *N* = 68; post: *N* = 40). **(d)** Overall satisfaction with the teaching model (*N* = 40).

Knowledge test scores were non-normally distributed. The median total score increased significantly from 60 (40, 80) before the intervention to 100 (80, 100) after the intervention (*P* < 0.001; *U* = 658.500; Table 4). Significant improvements were observed in four of the five knowledge domains, including HCC risk factors, early screening, metastatic patterns, and curative treatment. No significant difference was observed for HCC staging. Students also perceived the 4C-HOPE model as highly helpful for learning. Overall, 80.0% of students rated the model as “extremely helpful,” while 15.0% considered it “moderately helpful”. No student reported that the model was unhelpful (Figure 2a).

**Table 4:** Comparison of pre- and post-intervention knowledge test scores [*M* (*P*25, *P*75)].

Item	Pre-intervention (*N* = 68)	Post-intervention (*N* = 40)	*U* value	*P* value
Total score	60 (40, 80)	100 (80, 100)	658.500	<0.001*
Q1: HCC risk factors	20 (0, 20)	20 (20, 20)	1042.000	0.005*
Q2: Early screening for HCC	20 (0, 20)	20 (20, 20)	962.000	0.001*
Q3: Metastatic patterns of HCC	0 (0, 20)	20 (5, 20)	880.000	<0.001*
Q4: HCC staging	20 (0, 20)	20 (5, 20)	1200.00	0.209
Q5: Curative treatment for HCC	20 (0, 20)	20 (20, 20)	1104.000	0.034*

*P* values were calculated using the Mann–Whitney *U* test.

HCC, hepatocellular carcinoma.

*P < 0.05 indicates a statistically significant difference.

### 3.4 Learning interest and professional development

Learning interest in HCC improved significantly following the intervention. The proportion of students who were “very interested” increased from 50.0% before the intervention to 85.0% after the intervention (*P* < 0.001; *U* = 883.000; Figure 2b).

With respect to professional identity formation, the proportion of students who believed that physicians should provide holistic care for patients with advanced HCC increased from 89.71% to 95.0%, whereas the proportion favoring a more technically focused approach decreased from 10.29% to 5.0%. However, this difference did not reach statistical significance (*P* = 0.480).

Students’ willingness to apply positive psychology–based communication strategies shifted across response categories, although the overall difference was not statistically significant (*P* = 0.089; *U* = 1139.500) (Figure 2c). The proportion of students selecting “neutral” remained similar (4.4% versus 5.0%). The proportion reporting “willing” decreased from 35.3% to 17.5%, while the proportion selecting “very willing” rose from 60.3% to 77.5%.

### 3.5 Student satisfaction

Students reported high levels of satisfaction with the 4C-HOPE teaching model. Among post-intervention respondents, 87.5% were “very satisfied” and 12.5% were “satisfied” with the teaching experience. No student expressed dissatisfaction (Figure 2d). In response to an open-ended question regarding the teaching model, students provided generally positive feedback. Among the 19 students who responded, four students commented that the teaching was excellent, while 13 students indicated that the teaching model required no further improvement. In terms of constructive suggestions, one student noted verbatim: “Reduce the proportion of liver anatomy content and allocate more time to HCC diagnosis and staging,” while another stated: “More time should be allocated to the treatment section”.

### 3.6 Long-term follow-up outcomes

The mean theoretical examination score at clerkship completion was significantly higher in the 4C-HOPE group than in the comparison cohort who completed the conventional curriculum (81.49 ± 7.87 vs. 77.45 ± 8.58, *P* = 0.014; Table 5). These findings suggested that the benefits of the intervention may extend beyond the classroom and contribute to subsequent academic performance in clinical training.

**Table 5:** Comparison of oncology clerkship examination scores (M ± SD).

Examination	4C-HOPE Group (*N* = 36)	Control Group (*N* = 104)	*P* value
Total score	81.49 ± 7.87	77.45 ± 8.58	0.014*

*P* value was calculated using the independent samples *t*-test. *P < 0.05 indicates a statistically significant difference.

## 4 Discussion

This study examined the educational impact of the 4C-HOPE teaching model, a positive psychology–based approach integrated into undergraduate oncology education. The findings suggest that the model was associated with improvements in learning outcomes, learning interest, professional development, as well as sustained benefits in subsequent clerkship examination performance, while also receiving high levels of student satisfaction.

### 4.1 Improvement in learning outcomes

This study demonstrated a significant increase in HCC-related knowledge following the intervention, with median test scores increasing from 60 to 100 (*P* < 0.001). Improvements were observed across most content domains, indicating that the integration of positive psychology did not compromise—and may even have facilitated—academic learning. Notably, these benefits appeared to extend beyond the immediate classroom setting. During subsequent oncology clerkships, participants in the 4C-HOPE curriculum outperformed their peers who received the conventional curriculum on theoretical examinations, suggesting that the intervention fostered knowledge retention in subsequent clinical training.

Both objective performance and subjective student perceptions support the model's pedagogical value. One possible explanation is provided by Fredrickson's broaden-and-build theory, which proposes that positive emotions broaden individuals’ cognitive scope and facilitate information processing, learning interest, and resource building (21, 22). By incorporating patient narratives, survivorship experiences, and examples of therapeutic progress, the 4C-HOPE model may have elicited positive emotions and enhanced students’ learning interest, thereby facilitating knowledge retention. Nonetheless, the lack of significant improvement in HCC staging knowledge (*P* = 0.209) warrants attention. Given the concurrent use of three staging systems (AJCC, BCLC, and China Liver Cancer Staging), student difficulty in this area is understandable—a view corroborated by their feedback.

### 4.2 Enhancing learning interest

Oncology has been consistently perceived by medical students as an emotionally demanding and professionally challenging discipline, which may undermine learning motivation and career interest (23, 24). Traditional undergraduate oncology curricula tend to focus on disease-oriented knowledge delivery, which may reduce students’ learning interest (25).Consistent with this, students in the present study reported a strong sense of helplessness toward HCC before the intervention. Encouragingly, following implementation of the 4C-HOPE curriculum, we observed a marked improvement in students’ interest in learning about HCC (*P* < 0.001).

Positive psychological capital, consisting of hope, resilience, optimism, and efficacy (26), has been shown to positively influence medical students’ learning passion (27). Importantly, psychological capital is considered a developable psychological resource that can be enhanced through intentional interventions (28). The 4C-HOPE model was developed based on this rationale, incorporating educational elements that may cultivate positive psychological resources, such as efficacy, hope, and optimism, thereby potentially enhancing students’ engagement with oncology learning. Specifically, the Control stage aims to strengthen students’ sense of agency by highlighting modifiable cancer risk factors and preventive strategies. The Confidence stage fosters hope and resilience through updates on advances in cancer diagnosis and treatment. In line with this framework, accumulating evidence within positive psychology and medical education underscores the value of nurturing psychological resources instead of merely targeting the alleviation of negative emotions (29). Meanwhile, prior research indicates that medical students exhibit good receptiveness to embedding positive psychology principles within undergraduate medical training (30).

### 4.3 Promoting professional development

Medical students continue to express a need for greater attention to the psychosocial dimensions of cancer care (31). This need was also evident in our cohort, where nearly 70% of students desired practical strategies for instilling hope in their future patients. Following the intervention, more students endorsed holistic care as an essential component of oncology practice and expressed greater willingness to apply positive psychology–based communication strategies in clinical practice. Although these changes did not reach statistical significance, the observed trends suggest a shift toward more patient-centered attitudes and greater recognition of physicians’ psychosocial roles. Such changes are noteworthy given that burnout may originate during undergraduate medical training (32), whereas positive psychology–based education has been suggested to strengthen medical students’ clinical confidence and support long-term patient care (33).

These observed shifts toward patient-centered attitudes may be partly explained by the incorporation of patient illness narratives in the Compassion stage of the 4C-HOPE model. Narrative medicine emphasizes engaging with patients’ lived experiences and understanding illness beyond biomedical descriptions. Previous studies have shown that narrative-based approaches can enhance medical students’ empathy, reflective capacity, and patient-centered communication skills (34). In addition, narrative medicine has been associated with reduced clinician burnout, improved professional well-being, and greater confidence in clinical practice (35, 36). In the present study, patient narratives were used as educational materials to facilitate perspective-taking and deepen students’ understanding of patients’ emotional experiences rather than as psychological interventions. By integrating narrative learning with positive psychology, the Compassion stage may have helped students move beyond a predominantly disease-centered perspective toward a more holistic and patient-centered understanding of cancer care, potentially contributing to the observed changes in their perceptions of the physician's role.

Professional identity formation is widely recognized as a gradual and longitudinal process shaped through repeated educational and clinical experiences (37, 38). Therefore, it is not surprising that a single classroom-based intervention produced only modest short-term changes. Future integration of the 4C-HOPE model into clinical clerkships, simulation training, and longitudinal reflective activities may strengthen its impact on professional development over time.

### 4.4 Educational significance for oncology training

Medical education can negatively impact students’ mental health, Slavin et al. have demonstrated that preclinical curricular reforms could significantly reduce medical students’ stress levels (39). West et al. showed that both individual-focused and organizational interventions were associated with significant reductions in physician burnout, highlighting the importance of integrating resilience-building and psychological support strategies into medical education at an early stage (40). The 4C-HOPE curriculum, which integrates positive psychological capital components into the teaching of oncology risk factors, diagnosis, treatment, and prognosis, offers a promising pedagogical model that simultaneously enhanced students’ knowledge acquisition and learning interest.

### 4.5 Limitations and future prospects

This study has several limitations. First, the relatively small sample size (68 students in the pre-class survey and 40 in the post-class survey), together with the unmatched pre- and post-intervention design, may have reduced statistical power and introduced potential selection bias. Second, the attrition rate between the pre- and post-intervention surveys was substantial (41.2%). Although the primary reason for non-response was practical constraints rather than active refusal, we cannot exclude the possibility that students who were less engaged or had less favorable learning experiences was disproportionately absent. This may have introduced attrition bias, potentially leading to overestimation of the intervention effects. Future studies should consider implementing strategies to minimize missing data, such as offering multiple assessment time points or providing incentives for completion. Third, baseline equivalence between the two groups in the clerkship examination comparison could not be assessed. Thus, the observed difference in examination performance may have been affected by baseline differences or unmeasured confounding factors and should be interpreted with caution. Fourth, the knowledge assessment tool comprised only five multiple-choice items, which may not adequately capture the full breadth of students’ learning outcomes across the HCC chapter. Future studies could adopt a larger item pool or more comprehensive assessment strategies to better reflect students’ mastery of the material. Fifth, the questionnaire was self-developed based on a literature review for this exploratory study, and formal validation procedures were not conducted. The pre-intervention questionnaire showed relatively low internal consistency (Cronbach’s *α* = 0.621), likely due to its multidimensional nature covering diverse constructs at baseline. Future studies should refine the questionnaire through systematic validation to improve its validity and reliability. Sixth, long-term outcomes beyond this period—including clinical internship performance and enduring professional behaviors—remain unclear.

Future research should address these limitations through several directions. First, randomized controlled trials with larger sample sizes and control groups are needed to strengthen causal inference. Second, extended longitudinal studies tracking students into clinical internships and early career practice are required to evaluate the durability of educational effects on both clinical performance and professional development. Third, qualitative methods such as semi-structured interviews could be incorporated to explore students’ learning experiences and elucidate the mechanisms underlying cognitive and affective transformation. Finally, the applicability and generalizability of the 4C-HOPE model should be further examined across different oncology topics (e.g., lung and breast cancer) and diverse clinical training contexts, including clerkships and internships.

## 5 Conclusion

The 4C-HOPE teaching model is associated with significant improvements in knowledge acquisition and learning interest among undergraduate medical students, along with favorable trends in holistic care awareness and communication-related willingness. Improved performance during clinical clerkships further suggests potential benefits for subsequent clinical learning. These findings indicate that integrating positive psychology into undergraduate oncology education may offer a feasible approach to supporting both academic performance and early professional development.

## Data Availability

The raw data supporting the conclusions of this article will be made available by the authors, without undue reservation.
