# Supplementary material for: Integrating positive psychology into undergraduate oncology education: development and evaluation of the 4C-HOPE teaching model
Source: Front Med (Lausanne). 2026 Sep 9;13:1914104. doi: 10.3389/fmed.2026.1914104 (PMC13597455; doi:10.3389/fmed.2026.1914104)
Supplement: Supplementary file 1 [file Datasheet1.docx]

**《肝癌章节》测试题目及解析**

**1.肝癌的****常见高危因素不包括：**

A. 慢性乙型肝炎

B. 黄曲霉毒素

C长期酗酒

D. 药物性肝损伤（正确答案）

解析：慢性乙型肝炎、黄曲霉毒素和长期酗酒都是明确的肝癌高危因素，而药物性肝损伤虽然可能导致肝损伤，但不是肝癌的主要高危因素。

**2.对于高危人群，建议AFP联合下列哪个检查进行肝癌早期筛查：**

A.肝脏超声（正确答案）

B.增强CT进行三期扫描

C.增强MR，造影剂最好使用普美显

D.肝脏活检明确病理诊断

解析：AFP联合肝脏超声是肝癌早期筛查的标准方法，其他选项虽然也经常用于肝癌诊断，但不是常规筛查手段。

**3.癌与肉瘤的转移方式存在区别，肝细胞癌最常见的的转移方式是：**

A.淋巴转移

B.血行转移

C.肝内播散（正确答案）

D.种植转移

解析：肝细胞癌属于癌，但其最常见的转移方式是肝内播散，不是淋巴转移。

**4.影响中国肝癌分期因素不包括：**

A.肝功能 Child-Pugh 分级

B.肿瘤大小

C.肝外转移

D.全身状况

E. AFP（正确答案）

F.血管侵犯

解析：中国肝癌分期主要考虑患者全身状况、肝功能、肿瘤情况、血管侵犯等因素，不涉及AFP水平。AFP水平与肝癌筛查、诊断、判断疗效或复发具有重要意义，但与与分期无关。

**5.肝癌的根治性治疗手段不包括**

A.手术切除

B.肝移植

C.局部消融治疗

D.系统性药物治疗（正确答案）

解析：手术切除、肝移植和局部消融治疗是肝癌的三大根治性手段。

**HCC Chapter Test Questions and Standard Answer Key**

**1. The most common risk factor for hepatocellular carcinoma does NOT include:**

A. Chronic hepatitis B
B. Aflatoxin
C. Long-term alcohol abuse
D. Drug-induced liver injury (Correct Answer)

*Explanation:* Chronic hepatitis B, aflatoxin, and long-term alcohol abuse are all well-established high-risk factors for HCC, whereas drug-induced liver injury, although it may cause liver damage, is not a major risk factor for HCC.

**2. For high-risk populations, AFP combined with which of the following examinations is recommended for early HCC screening:**

A. Liver ultrasound (Correct Answer)
B. Contrast-enhanced three-phase CT
C. Contrast-enhanced MRI, preferably with Primovist
D. Liver biopsy for pathological diagnosis

*Explanation:* AFP combined with liver ultrasound is the standard method for early HCC screening. The other options, although commonly used for HCC diagnosis, are not routine screening tools.

**3. The metastatic patterns of carcinoma and sarcoma differ. The most common metastatic route of hepatocellular carcinoma is:**

A. Lymphatic metastasis
B. Hematogenous metastasis
C. Intrahepatic spread (Correct Answer)
D. Implantation metastasis

*Explanation:* Hepatocellular carcinoma is a carcinoma, but its most common metastatic route is intrahepatic spread, not lymphatic metastasis.

**4. Which of the following is NOT a factor in the China Liver Cancer Staging (CNLC) system:**

A. Child-Pugh classification
B. Tumor size
C. Extrahepatic metastasis
D. Performance status
E. AFP (Correct Answer)
F. Vascular invasion

*Explanation:* The China Liver Cancer Staging system primarily considers the patient's performance status, liver function, tumor characteristics, and vascular invasion, and does not include AFP levels. AFP is important for screening, diagnosis, treatment response assessment, and recurrence monitoring, but is not relevant to staging.

**5. Curative treatment modalities for hepatocellular carcinoma do NOT include:**

A. Surgical resection
B. Liver transplantation
C. Local ablation therapy
D. Systemic drug therapy (Correct Answer)

*Explanation:* Surgical resection, liver transplantation, and local ablation therapy are the three major curative treatment modalities for HCC. Systemic drug therapy is not considered a curative treatment.
